# From experience to a learning health system: peer-to-peer perspectives and implications for healthcare navigation in Alberta, Canada

**DOI:** 10.3389/frhs.2025.1642188

**Published:** 2025-10-17

**Authors:** Fakhriyya Aghabayli, Ingrid Nielssen, Luz Aida Zapata-Cardona, Safa Ahmed, Chisom Ezemenahi, Sukhwant Parmar, Boman Fahimi, Maryem Hamid Khan, Huda Hamid Khan, Naxhielli Donaji Mendez Muniz, Ugochukwu Osigwe, Shaziah Zaidi, Xingye Shi, Kiran Nabil, Paul Fairie, Maria Jose Santana

**Affiliations:** ^1^ Alberta Strategy for Patient-Oriented Research Support Unit, Calgary/Edmonton, AB, Canada; ^2^ Cumming School of Medicine, University of Calgary, Calgary, AB, Canada; ^3^ Department of Continuing Education, University of Calgary, Calgary, AB, Canada; ^4^ Department of Community Health Sciences, Cumming School of Medicine, University of Calgary, Calgary, AB, Canada; ^5^ Department of Pediatrics, Cumming School of Medicine, University of Calgary, Calgary, AB, Canada

**Keywords:** person-centered care, patient navigation, learning health systems, patient engagement, peer-to-peer research, healthcare navigation

## Abstract

**Background:**

Healthcare navigation services help individuals access timely and appropriate care within complex health systems, particularly those facing systemic and equity-related barriers. Understanding navigation experiences is essential to addressing service gaps and improving health outcomes. This study sought to examine the lived experiences of navigation in Alberta to identify inequities within existing programs and to provide recommendations for strengthening person-centered navigation within a learning health system framework.

**Materials:**

This was a qualitative, peer-to-peer, patient-oriented research study. The study design followed the Patient and Community Engagement Research process of SET-COLLECT-REFLECT. The SET phase engaged patient and public partners in discussions to co-design the research question and the study design. The COLLECT phase included focus groups and interviews with adult residents in Alberta who had been navigated (*n* = 13) and those who had experience as healthcare navigators (*n* = 13) in the Alberta healthcare system. The data were thematically analyzed, identifying key themes and subthemes. The REFLECT phase ran two focus groups with COLLECT participants for member checking. This approach yielded the recommendations.

**Results:**

Of the 26 participants, over 75% were women (77% of the Navigated group and 75% of the Navigator group) aged 41–50 years old. Half of those in the Navigator group had provided their service for more than 5 years and had received specialized training in healthcare navigation. The following themes were identified: (1) participants’ situations and circumstances, (2) navigation experiences, (3) perspectives, (4) need for healthcare navigators, (5) the navigator role, (6) current best practices and challenges, and (7) training and support. Five recommendations included expanding the scope and enhancing awareness of navigation programs with a personalized approach and embedded evaluation and developing and formalizing navigation training programs.

**Conclusion:**

This study identified gaps and opportunities in healthcare navigation programs from both navigator and navigated perspectives. The findings provide patient-centered recommendations to strengthen navigation services and their integration into Alberta's learning health system that can enhance equitable access, healthcare experiences, and outcomes.

## Introduction

Healthcare systems have grown increasingly complex due to specialization and expanding care pathways, creating significant challenges for individuals attempting to navigate care on their own (1). These challenges are particularly pronounced for people living with new diagnoses, those living with chronic and complex conditions, and those facing systemic barriers rooted in structural and social inequities. Such inequities, including those related to income, culture, language, geography, and disability, limit equitable access to timely, appropriate, and person-centered care. In response, various navigation supports and services have been developed, including in Alberta, Canada, the focus of this study.

The concept of patient navigation was first introduced in 1990 by Dr. Harold Freeman to reduce inequities in cancer care access among racialized and marginalized populations in Harlem, USA (2). Since then, navigation has evolved into a global strategy addressing a wide range of conditions and needs (3). In Alberta, navigation programs currently serve diverse populations across cancer care, diabetes, mental health, disability, life transitions, and newcomer support. However, the varied definitions and applications of “patient navigation” have led to confusion, hindering efforts to evaluate their effectiveness, improve equity in service delivery, and standardize navigator training.

More recently, the term “healthcare navigation” has been adopted to describe a broader spectrum of services, ranging from community-based wellness supports to specialized disease care. Reid et al. delineate between lay navigators (e.g., peers, community health workers, and informal caregivers) who share lived experiences with those they support, and professional patient navigators (e.g., nurse navigators, care coordinators, and diabetes educators) with clinical expertise (4). Both play important roles in addressing inequities by bridging gaps in access, fostering trust, and supporting culturally responsive, person-centered care.

In Alberta, navigation services span diverse aspects of healthcare such as cancer, diabetes, mental health, disability, life transitions, and newcomer support. Understanding healthcare navigation through a health equity lens is essential to identifying barriers, addressing gaps, and advancing system-level changes that promote fairness in access and outcomes (3, 5). This study sought to examine the lived experiences of healthcare navigation in Alberta to identify inequities within existing programs and provide recommendations for strengthening person-centered navigation within a learning health system framework.

## Materials and methods

To address the objective, this peer-to-peer patient-oriented qualitative study was conducted. People with lived experience were meaningfully engaged throughout the design, development, and dissemination phases of the research process to inform more person-centered healthcare policy and practice.

A literature review and an environmental scan of existing healthcare navigation programs in Alberta provided the evidence base for this study (described elsewhere). The methodology used a participatory action research approach (6) based on the Patient and Community Engagement Research (PaCER) process that includes three phases: SET, COLLECT, and REFLECT (7–9).

### The PaCER program

PaCER is a 1-year experiential-based participatory research training program supervised by the Alberta Strategy for Patient-Oriented Research (AbSPORU) SUPPORT Unit, Patient Engagement Team (10), in partnership with the Continuing Education program at the University of Calgary (7–9).

The study was conducted during an 80-hour research project as part of the PaCER program by a team of PaCER students that comprised individuals from diverse ethno-cultural, academic, professional, and research backgrounds and with distinct lived experiences of being navigated and/or as navigators in the Alberta healthcare system. These students were fluent in 10 languages, including Arabic, Azerbaijani, Dari, Hindi, Mandarin, Pashto, Punjabi, Russian, Spanish, and Urdu. The students were divided into two groups of six members each, namely, the Navigated and Navigator groups, to gain a focused understanding of the unique experiences and insights from each of the two perspectives. These were then brought together to offer a more comprehensive and holistic overview of the current contexts of healthcare navigation in Alberta.

**Figure 1:**
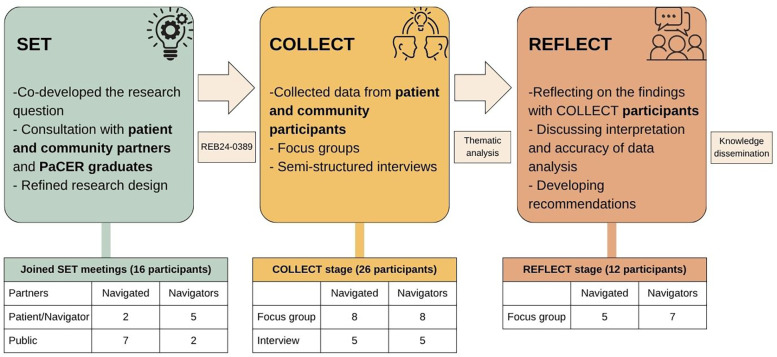
The PaCER process.

The PaCER process (Figure 1) consisted of the following three phases:

1. SET: Both the Navigator and Navigated groups held discussions with those with lived experience of healthcare navigation and public partners to refine the research question, research design, and focus group and interview question guides. The research protocol was approved by the University of Calgary Conjoint Health Research Ethics Board (REB24-0389).
2. COLLECT: Participant recruitment for the focus groups and interviews started in April 2024. Participants were adults, residents of Alberta, and with experience of being navigated in the Alberta healthcare system or had experience navigating people in the Alberta healthcare system. Recruitment was conducted through purposive sampling with convenience selection of participants. The Navigator and Navigated groups recruited participants for their focus groups and interviews using unique recruitment posters (see Supplementary Material). Recruitment posters were shared with the organizations identified through the environmental scan of healthcare navigation organizations in Alberta and through the team members’ community connections, the Albertans4HealthResearch.ca network (11), and community-based social media platforms, including chronic disease support groups. This supported diversity in socioeconomic, cultural, and navigation experiences. In addition, to be inclusive, the participants were asked preferences regarding languages, delivery mode (online or in person), and scheduling of focus groups or interviews. Data from the COLLECT stage were thematically analyzed, and key recommendations were synthesized based on developed themes. Data saturation in this study was determined when no new themes emerged from the data analysis, indicating that sufficient information had been collected to address the research objectives.
3. REFLECT: The COLLECT focus group and interview participants were invited to the REFLECT focus groups for member checking (12) to ensure accuracy of interpretation and findings. Recommendations were renewed based on the REFLECT participants’ comments and suggestions.

### Data collection and analysis

All the participants were asked to complete a sociodemographic electronic Qualtrics survey that included questions about characteristics unique to each group, e.g., number of patients and hours offering navigation services per week for the Navigator group and number of years living in Canada for the Navigated group. All the participants were offered a prepaid gift card ($25 CAD) in appreciation for their participation in each of the COLLECT and REFLECT focus groups and interviews they joined.

Interpretation services were offered in the 10 different languages previously mentioned to the focus groups and interview participants. All the focus groups and interviews were held online via the Zoom platform. All the participants provided oral or electronic consent to participate using a REB-approved informed consent form. The participants were asked for permission to video record the focus groups or interviews for notetaking purposes only. The focus groups were 2 h long. At least three team members conducted the focus groups, with one facilitating the session, and the other two managing the chat, taking notes, and supporting participants with sign-on or Zoom issues. Participants who were unable or uncomfortable joining the focus groups shared their experiences in a 1-h online semi-structured interview. Two team members conducted the interviews—one asked the questions and the other took detailed notes. Participation of multiple team members at the focus groups and interviews was a priority to allow diverse points of view and limit personal bias. The focus groups and interview question guides contained the same questions. (See Supplementary Table 5 for the Navigated Focus Group Question Guide and Supplementary Table 6 for the Navigator Focus Group Question Guide).

Zoom transcripts of the focus groups and interviews were de-identified, cleaned and sorted, and arranged into Excel files. The data were then analyzed using the six-step thematic analysis approach as described by Braun and Clarke (13), including (1) familiarization with the data, (2) generating initial codes, (3) searching for themes, (4) reviewing themes, (5) defining and naming themes, and (6) writing the report. The transcripts were collectively analyzed. These discussions encouraged critical reflection on each member's interpretations and provided opportunities to challenge underlying assumptions and preconceptions. After each member individually coded the first focus group, each team created a code book that included a brief description of the codes as they related to the context of the study. These code books were then applied to the remaining focus group and interview data, adding additional codes as they emerged. Codes were consolidated into categories and then organized into themes and key recommendations.

## Results

### Demographics

Table 1 depicts the participants’ sociodemographic characteristics. Of the 26 participants, 13 were people with lived experience of being navigated in the Alberta healthcare system, and the other 13 were healthcare navigators. The navigators had experience in serving in multiple areas (rural and urban) and covering a wide range of health conditions, including domestic violence, and all spoke at least one additional language to English. The navigated participants represented a wide range of socioethnic backgrounds, languages, and health conditions.

**Table 1:** Participants’ sociodemographic characteristics.

Characteristic	Number (%) of participants in the Navigated group; *n* = 13 participants	Number (%) of participants in the Navigator group; *n* = 13 participants
Gender/Sex		
Male	3 (23)	2 (17)
Female	10 (77)	9 (75)
Age group		
31–40	3 (23)	4 (33)
41–50	6 (46)	4 (33)
51–60	2 (15)	3 (25)
>60	2 (15)	1 (8)
Ethnic background		
Black		1 (8)
Caucasian	4 (30)	2 (17)
South Asian	6 (46)	3 (25)
Arab		2 (17)
Latin American		3 (25)
East African	1 (8)	1 (8)
East Asian	1 (8)	
Hungarian	1 (8)	
Length of time in Canada		
Born here	2 (15)	N/A
Less than 5 years	1 (8)	
5 years or more	8 (62)	
Prefer not to answer	2 (15)	
What is your immigration status?		
Citizen	11 (85)	N/A
Permanent resident	2 (15)	
Work permit	0	
Visitor	0	
Other		
Where was the navigation service provided?		
Urban Alberta	12 (92)	11 (92)
Rural Alberta	1 (8)	3 (25)
Mode of delivery of navigation service		
In-person	1 (8)	9 (75)
Online	9 (69)	6 (50)
Phone	3 (23)	4 (33)
Primary language navigation service was offered in		
English	5 (38)	5 (42)
Other languages	8 (62)	6 (50)
Arabic		2 (17)
Spanish		1 (8)
Punjabi	2	
Hungarian	1	
Mandarin	1	
Specific health conditions or populations that required navigation services/specific health conditions or populations served		
Cancer	2 (15)	1 (8)
Diabetes	1 (8)	2 (17)
Indigenous populations	2 (15)	1 (8)
Mental health	3 (24)	4 (33)
Others	1 (8)	7 (67)
All patients and populations	1 (8)	
Disabilities	2 (15)	
Life transitions	1 (8)	
New immigrants		
Stroke		
Pregnancy and hernia		
Cervical cancer		
Heart condition		
Diabetes, cardiovascular disease, cancer, eye disease		
Duration or length of time using/providing navigation services		
<1 year	3 (23)	2 (17)
1–3 years	5 (38)	4 (33)
3–5 years	2 (16)	6 (50)
>5 years	3 (23)	
Number of patients assisted in a week		
0–5	N/A	4 (33)
5–10		1 (8)
10–15		4 (33)
>15		2 (17)
Financial compensation for receiving/providing navigation services	For receiving navigation services	For providing navigation services
Yes	0	5 (42)
No	13 (100)	4 (33)
Sometimes	0	3 (25)
What organization were you receiving navigation services from?		
Family doctor's office	2 (15)	N/A
Family/relatives	3 (24)	
Friends	2 (15)	
Alberta Health Services	2 (15)	
Tom Baker Cancer Center	1 (8)	
Hospital clinic	2 (15)	
Diabetes educator	1 (8)	
Specialized training in healthcare navigation		
Yes	N/A	7 (58)
No		5 (42)

The majority of the participants in both groups were female (77% of the Navigated group and 75% of the Navigator group). The largest age group among the participants in the Navigated group was 41–50 years (46%), while those in the Navigator group were distributed across 41–50 years (33%) and 31–40 years (33%). The majority of the navigation services were provided and received in Alberta's urban settings (92% in both groups).

The participants in the Navigated group most often accessed services online (69%) or by phone (23%), whereas those in the Navigator group primarily delivered services in person (75%) or online (50%). A substantial proportion of the participants spoke a language other than English (62% of the Navigated group and 50% of the Navigator group). While all participants in the Navigator group were fluent in English, two participants in the Navigated group who spoke Punjabi reported limited English proficiency.

Half of the participants in the Navigator group (50%) had more than 5 years of experience, while 38% of the participants in the Navigated group had received navigation services for 1–3 years. All participants in the Navigated group accessed services free of charge, and a majority of those in the Navigator group (42%) received compensation for their work. In addition, 58% of the participants in the Navigator group reported receiving specialized training in healthcare navigation.

### Thematic analysis

The themes identified by both groups reflected the complexity of the healthcare system and shed light on gaps in healthcare navigation services and navigator training programs. The themes from the Navigated group reflected the participants’ unique situations and circumstances, and their perspectives about being navigated in the Alberta healthcare system. The participants in the Navigated group described improved access to care, care experiences, and health outcomes because of their navigation. The themes from the Navigator group centered on the need for healthcare navigators, the navigator role, current best practices, challenges in working as a healthcare navigator, and insights and recommendations to improve training and support for healthcare navigators. Tables 2, 3 outline the themes, subthemes, and exemplar quotes.

**Table 2:** Themes from the Navigated group.

Theme 1: Participants' situation and circumstances
Subthemes
1.1 Healthcare system complexity: Perceived and/or experienced barriers and challenges in accessing primary healthcare, disease diagnosis, and/or treatment. * *“That's what I’ve noticed for myself. Yeah, so I would say overall like the most frustrating parts I found in the system is like the wait times for connecting with the specialists and sometimes like the communication between different parts of the system or not there.” (FG2-P1) 1.2 Personal situation: Participants’ characteristics and circumstances when seeking and receiving healthcare navigation services (including the following social determinants of health: economic, educational background, and other factors). “Those were my struggling years. I remember them as my struggling years here because I was just alone with a newborn baby, a 5-year-old and me.” (Int5)
Theme 2: Navigation experience
Subthemes 2.1 Awareness of navigation services: Knowledge and understanding of the availability, accessibility, and eligibility of healthcare navigation services, including those used, and recommendations about increasing awareness. “I didn’t even realize that a patient navigator service existed. I’ve been Type 1 diabetic all my life.” (FG1-P1) 2.2 Navigation services received: Participants’ perspective on the access, availability, modes, and means used to provide the navigation service. “Oh, yeah, my daughter helped me. But my son in law, he makes me appointments and takes me to the doctor. I must go for checkups because I have pacemaker. He takes me there, so I don’t have a problem.” (FG3-P1) 2.3 Participants' experience of being navigated: Participants' feedback on how the service was delivered and whether it was helpful or not to the participant. “I guess everybody who is diagnosed with cancer gets a nurse navigator but in my opinion it's useless because at that stage you don’t, you don’t know what specific type of cancer you have … But after I had my surgery, everything changed, but I never spoke to nurse navigator again.” (FG1-P2)
Theme 3: Participants' perspectives
Subthemes 3.1 Ideal navigator: Characteristics of the participants' ideal navigator (education, training, gender, language, cultural, professional, shared lived experience backgrounds, and competencies). “I would like that person to be trust, someone with degree in healthcare, and who was lived here for a long period of time. Gender doesn’t matter to me. But if he is the same culture, you feel that kind of connection I would say. So same cultural background, lived experience here and was lived here for you know good amount of time.” (Inv1) 3.2 Ideal navigation service: Vision of specific services and delivery modes for optimal navigation and recommendations to achieve it (including suggestions for condition-specific navigation programs). “Yeah, or like a finding a plan to like do something in the interim while you are waiting for that specialist, like what you should be doing, what maybe can help you.” (FG2-P1)

**Table 3:** Themes from the Navigator group.

Theme 1: Need for healthcare navigators.
Subthemes 1.1 Complexities of the healthcare system: Complexities and disparities in care delivery within the healthcare system (challenges and barriers to equitable care experience and health outcomes affected by individual social determinants of health). “Yeah, so what I think is, things that are done from an administrative and policy perspective don’t really know how it works on the ground. So, I think they need to try it. Like, okay, you have done the administrative part, you’ve put the rules but you need to see on the ground how it really works.” (Int1–331) 1.2 Barriers and gaps in care: Gaps in coordination of care across multiple healthcare professionals, clinics, and organizations (including those due to regular updates and changes to policies and practices). “I know they put the policies with the best intentions; that is a definite! But then, can they be implemented as they have it in their mind? A lot of gaps arise, a lot of issues happen, because you might envision something but then when you actually come to implementing, it doesn’t work.” (FG1-P3) 1.3 Multiplicity of support, services, and resources: Existing resources and support within the Alberta healthcare and social services system (including challenges in identifying, assessing, and accessing the healthcare system for recent immigrants with a language barrier when receiving a devastating diagnosis). “Now we have pharmacist educators, and I also engage the pharmacist with me. I found out that there are so many educational platforms at the pharmacy in the different setups in the hospitals, but when it comes to community awareness, there was a little bit of lacking in education for specific communities.” (Int5)
Theme 2: The navigator role
Subthemes 2.1 Value of the navigator role: Positive impact of the navigators in enhancing their clients’ ability to bridge gaps in their care and better navigate the complexities of the healthcare system (navigation successes, examples of clients’ gratitude stories). “She was alone here, so she gave birth here. She was all alone. Then, my organization helped her. I personally also helped her. We took care of her and the baby and then she was able to pass through all that difficult time. And then, she was very thankful because with all the help, she was able to pass through that experience. Otherwise, it would have been very traumatic for her, being very alone here in Calgary having no one around, and she was not able to involve anyone in her care. She was not familiar with the health care system.” (FG3–94, 95). 2.2 Motivation to become a navigator: Participants’ inspiration and motivation to become navigators (desire for others to have better healthcare experiences than what they had and a passion to help people, especially those marginalized in healthcare access). “You know, I’m a child of immigrants. So, you know, what happened when my parents were trying to navigate this all on their own and figure it all out? So that's kind of my motivation.” (FG1-P1) 2.3 Scope and description of navigator role: Description of the services the participants offered as part of their navigator role. “I am able to help in whatever health situation somebody presents to me with, but my biggest areas of specialty, I help people who are kind of at life transitions, Elderly people moving into retirement, retirement transitioning to long-term care. I help people who have been newly diagnosed with chronic disease, I help families who are seeking mental health support for their children whether it be a newly diagnosed child or for themselves as a late diagnosed adult with a mental health issue.” (FG1-P1) 2.4 Navigator–client relationship: Participants’ relationship and rapport with their clients. “It's always very rewarding now and then. She left back home and even now, she sends pictures of her baby growing up. Recently, the baby was one year old, and she sent me a video from his birthday celebration. It's always very rewarding and positive to see people growing and realizing at some point in their lives that you supported them.” (FG3-P1)
Theme 3: Current best practices and challenges in the navigator role
Subthemes 3.1 Systemic barriers and limitations to the navigator role: Systemic, institutional, and organizational limitations and obstructions to the navigators’ role. “Even for myself, when I was trying to call my doctor, there's so many rules about patient information security that you have to bypass. And I think if you’re a navigator and then you’re asked to be, you have to have all these permissions. Having the patient authorize you to speak on their behalf, be more readily available and maybe in the system, like how they do it in the bank—‘This person is authorized at all times to speak on their behalf. to advocate and ask questions’ that's not readily available in Alberta health services. And even in my case, when I was trying to communicate with my own doctor, but I was abroad. There was no way to break that protocol of not speaking with people who were calling internationally.” (Int1, 97–99) 3.2 Personal challenges that the navigators experienced: Personal limitations and challenges in delivering navigation services to clients [including moral and ethical dilemmas (medical expertise conflicting with the navigator’s advice)]. “Wait lists is a huge barrier in the mental health and addictions field right now. There's wait lists for everything, we’re constantly seeing funding cuts all across the board, which is making wait lists even worse. These kinds of things have definitely been a big challenge that I have faced in supporting families and getting help as quickly as possible.” (Int2, 90–92) 3.3 Importance of shared culture, language, background, and medical and lived experience of a health condition: Effectiveness of shared culture, language, and background between health navigators and clients in healthcare navigation and enhancement of person-centered care delivery. “But from my experience, from what I have felt and the feedback I received, especially from women, who are newly immigrants. I feel like they open up more and they feel more comfortable with a female. It's just … that's what they’re used to even back home from cultural perspective. So when they do come here and if they feel like, oh, she speaks the same language, and she is from the same gender; they sort of open up more. They can joke more, they feel more comfortable. They’re able to express more and be more vulnerable than when the interpreter or the navigator is a gentleman, they might, you know, be shy from a cultural perspective.” (FG-1, 316–317). 3.4 Need to formalize the navigator role and profession: Structures and frameworks for consistency in professional and ethical standards of practice, language, and delivery. “We don’t have standards of practice for navigators or even like a standard requirement for an education level or anything like that. It would really help the effect of health navigation if there was a standard of practice. Navigation is so tough because like you even said that we Navigators navigate for so many different things.” (Int2)
Theme 4: Training and support for healthcare navigators
Subthemes 4.1 Existing training: Training programs and workshops participants attended to support their role as navigators. “At work, I have been trained in navigation of patients. At the volunteer resource center, we have a huge booklet that we have to go through and we’re assessed. And we have annual staff education that we have to go through, to keep our role up to date in our minds and refresh our knowledge—how to use the wheelchair and how to talk to people and, you know, mental health, Indigenous awareness - all kinds of supports on how to be the best we can be in our role.” (Int4) 4.2 Ideal training for the navigator role: Suggested helpful training and professional development for improving navigators’ work (including more comprehensive training on the resources and services available to clients). “It has to be accredited. It has to, it has to like give a credit, proper training. And to extend it in different languages too…” (FG3-P2, 182–183) 4.3 Community of practice for navigators: Establishing communities of practice to support flexibility and personalized navigation for vulnerable populations (mental health, addiction, and refugees). “So, I think there has to be a level of trust in navigators, and really any health care professional. That people understand that they know they got this job because they know what they’re doing. To restrict them by standardization to a point, yes, but to put too many protocols in place for them will just create another silo that healthcare doesn’t need. We need navigators that can operate a little more freely than what the rest of the system is doing because that's why we need them right now.” (FG-1 406, 407). 4.4 Professional support: Helpful professional support to assist navigators to work in professional and healthy ways (including financial and mental health support). “In Alberta Health Services, there is a full, support system for you. If you are passing through anything like that, you can get a counselor as well and similarly with my other two organizations, you can have mental health counselors, and you are allowed to have just a day off for your mental health. Yeah, so if you think you are not feeling well or you think that you are exhausted mentally so you can get a paid (day) off.” (FG3-P1)

The Navigated group developed three themes: (1) participants’ situations and circumstances, (2) navigation experiences, and (3) perspectives.

1. Participant's situations and circumstances. The context in which navigation happened.
  “… well, I came from New Zealand, so it's similar health system …. Yeah, but I also had an issue with. … I, didn’t, wasn’t originally recognizing my, low blood sugars and I actually collapsed on one of the train stations downtown.” (FGD2-P2).
2. Navigation experiences. The participants’ unique perspectives and insights on their navigation experience(s).
  “Sometimes the needed language support. And having someone there who could speak on their behalf. Was helpful or like help them communicate or understand what they were being told by the medical professionals about like the kind of care that they needed.” (FGD2-P1).
3. Participant's perspectives. The participant's point of view about an ideal navigator and ideal navigation services.
  “Yeah. I feel like being a male or female is not important. So who has some knowledge, a person who can speak, our own language, participant one is saying, so language is very important and who can understand you can put themselves at in your position.” (FG-P1).

The Navigator group developed the following four themes:

1. The need for healthcare navigators. Social, systemic, and structural factors that define the need for healthcare navigators in Alberta.
  “In a lot of cases, once the treatment is completed, there isn’t necessarily a follow up, you know, in terms of, dialog with the patient. Lot of cases they felt feel or they feel as if they are left. And nobody nobody's following them.” (FG2-P1).
2. The navigator role. Participants’ motivations for becoming a navigator.
  “You know, I’m a child of immigrants. So, you know, what happened when my parents were trying to navigate this all on their own and figure it all out? So that's kind of my motivation.” (FG1-P1).
3. Current best practices and challenges. Effective strategies, gaps, and possible improvements in existing navigation service delivery in Alberta.
  “We don’t have standards of practice for navigators or even like a standard requirement for an education level or anything like that. It would really help the effect of health navigation if there was a standard of practice. Navigation is so tough because like you even said that we Navigators navigate for so many different things.” (Int2).
4. Training and support. Existing and proposed training programs, workshops, and professional development enhancing navigators’ work.
  “It has to be accredited. It has to, it has to like give a credit, proper training. And to extend it in different languages too…” (FG3-P2).

### Recommendations

The recommendations were derived from the data collected during the COLLECT and REFLECT phases. Five recommendations included expanding the scope and enhancing awareness of navigation programs, integrating more person-centered approaches, and embedding evaluation into the development and delivery of service and training programs. All the participants proposed more navigation service advocacy and flexible access, especially for vulnerable populations seeking timely mental health support. Including specialized interpretation and translation services to newcomer and immigrant populations was also deemed crucial, reflecting that English was a second language for the majority of the participants in both the Navigated and Navigator groups. The participants also emphasized the need for adequate financial compensation and mental health services for navigators to ensure ethical and sustainable support to significantly enhance navigation effectiveness and outcomes for both navigators and their clients.

The participants in the Navigator group described how often the lack of formal recognition and systemic limitations affected the scope of support they could provide to their clients. The navigators suggested formalizing the navigator role with flexible guidelines and best practices, while maintaining flexibility for personalized service delivery tailored to the unique needs of each client. As 58% of the participants in the Navigator group reported having a professional training in healthcare navigation, they identified a need for more comprehensive and professional training that covers both broad professional and ethics training for the navigator role and training specific to the health conditions and populations their clients represent.

The participants in the Navigated group highlighted the need to improve awareness and communication about navigation programs. They agreed that primary care clinics and emergency departments are crucial entry points to the healthcare system, making them ideal sources for accessing navigation information. Considering that the majority of the participants in the Navigated group used online navigation services, they also suggested improving the user interface of the Alberta Health Services website to a more patient-centered design, enabling the public to better access and navigate the extensive information available. In addition, immigration and refugee service providers in Alberta, along with immigration websites and entry points to Alberta, such as airports, were highlighted as strategic locations for disseminating information on accessing healthcare navigation services. The participants in the Navigated group emphasized the need to develop peer navigator services that acknowledge the unique role that peer navigators play by validating their feelings and sharing their experience in navigating certain conditions.

Furthermore, the participants highlighted the necessity of professional support for navigators, advocating for improved financial compensation (only 42% of the participants in the Navigator group were financially compensated for their services) and mental health services to ensure that navigators can fulfill their roles ethically and sustainably. Addressing these needs could significantly enhance the effectiveness of navigation services, ultimately leading to better outcomes for both navigators and the clients they serve. Finally, continuous evaluation of healthcare navigation services and training programs is integral to allow learning health systems (14) to adapt to evolving healthcare needs and ensure effective service delivery. Table 4 outlines the recommendations.

**Table 4:** Recommendations.

Recommendations to improve healthcare navigation service program delivery and navigator training in Alberta, Canada
1. Continue to expand the scope of formalized healthcare navigation service programs and the navigator role in Alberta to include additional health and wellness conditions. This can ensure navigation services are available throughout each unique patient diagnosis and care journey, especially for vulnerable groups such as those requiring cancer care, disability care, mental health services, emergency care, and transitional care (from acute to community care) and for care for children and the elderly, pregnant women, rural populations, and newcomers and refugees. “So which areas or a specific population do you believe needs more navigation services?” (Navigator TM) “Actually, all areas. But if you ask in particular, I think it's the geriatric one.” (Navigator Int 5) “…at the beginning of all of that, it's a very stressful time and you just want to talk to somebody who is going to help you and tell you and give you as much information as possible, right?” (Navigated, FGD1-P2)
2. Enhance awareness of Alberta-based healthcare navigation services through infographics, social media, community events, and educational sessions that include detailed information about eligibility, health conditions services are available for, timelines, and any costs involved in accessing navigation services. These should be available at patient, community member, and caregiver contact points with the healthcare system, such as family physicians’ offices, walk-in clinics, emergency rooms, pharmacies, and laboratory services. Furthermore, ensure online links for navigation services are prominently displayed on the main pages of health and social care websites and newcomer entry points. “I always Well, since starting this role, I’ve said a lot that the mental health and addictions field really sucks at marketing themselves, and people don’t know what programs are out there and what is around.” (Navigator Int 2) “And I know it would be too difficult, probably to do, but some way of like when you arrive in the country, you get a package with this is what you need to do.” (Navigated FGD2-P2)
3. Continue to develop and formalize training for the distinct roles of patient and lay navigators in Alberta, Canada, by incorporating adaptable guidelines and evidence-based best practices. Accredited patient navigator training programs could include core courses on patient navigation, professional development and ethical conduct, service and support awareness, cultural and linguistic training, and disease-specific modules. Support the development of a community of practice for conditions with unique or more sensitive navigation needs, such as mental health, addiction, and refugee support. “Actually, I believe health navigation services are quite ambiguous in Alberta. It should be a regulated profession.” (Navigator FG3-P1) “Yeah, definitely like, there should be proper training and proper recognition of the role.” (Navigator FG3-P1)
4. Ensure healthcare navigation remains person- and family-centered by always retaining a personalized approach to service delivery that is responsive to the unique needs and preferences of both the navigators and patients. Collaborate with healthcare providers, social workers, palliative care teams, and mental health and emotional support services to identify and access timely navigation services and follow-up that is tailored to the complexity of individual health conditions and circumstances. As much as possible, navigators should share similar languages, cultural backgrounds, and lived experiences with those they are navigating. “Well, first of all, I totally agree with FG1P1. It reminded me of a quote, you know, like when I used to read that quote that treat the patient as a person, not as a patient.” (Navigator FG1-P2) “So who has some knowledge, a person who can speak, our own language, participant one is saying, so language is very important and who can understand you can put themselves at in your position.” (Navigated FG4-P1) “…having all whose soft skills and being educated and having lived experience. That would be helpful for any newcomer.” (Navigated Int 1)
5. Program evaluation should be embedded in navigator training and navigation service delivery plans and be informed by those impacted by these programs. Integrate patient, community, and caregiver engagement through a learning health system model to ensure navigation training programs and navigation service delivery models are evolving and meeting increasingly complex social and healthcare system challenges. “We have to connect the supply and the demand. You know, the health care navigator with the patients and family, care givers and people in Alberta that need the navigation.” (Navigator Int4) “…I say that is sometimes you just need some listening ears. And that is the most important thing in your life that someone is listening to you.” (Navigated Int 5)

## Discussion

This study offers practical person-centered recommendations to improve existing healthcare navigation programs. Integrating these recommendations into learning health system initiatives in Alberta will support more equitable access to healthcare services and treatments and improve public and patient healthcare experiences and health outcomes for all populations.

**Figure 2:**
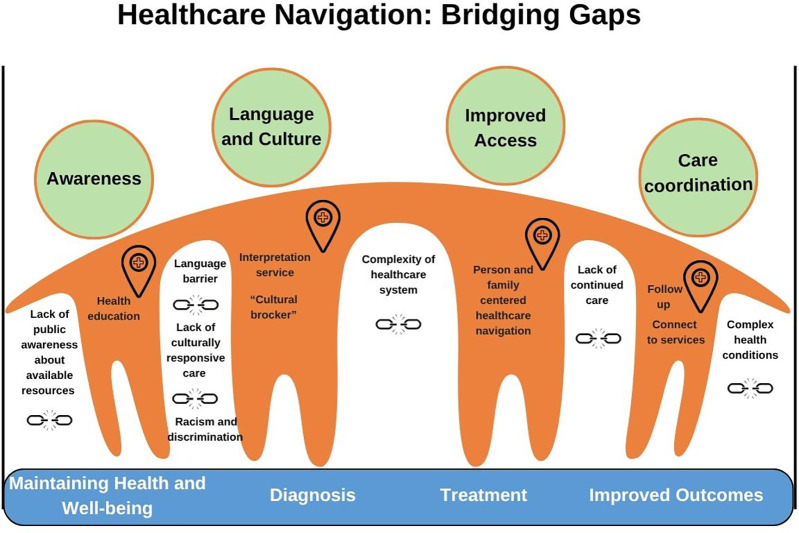
Healthcare navigation: bridging gaps.

This study confirmed that healthcare navigation is crucial for addressing gaps and barriers in accessing healthcare services (Figure 2), thereby improving one’s healthcare experience and health outcomes, especially for those facing systemic and other healthcare equity-denying barriers (3, 15–19). Several barriers to accessing appropriate and timely care, including the complexities of the healthcare system, can create gaps in an individual's healthcare journey, potentially causing them to fall through the cracks and negatively impacting their health outcomes. Challenges such as long wait times for specialist appointments, convoluted referral processes involving multiple healthcare professionals, and transportation issues are notable examples. Language barriers and a lack of culturally responsive care further complicate access to necessary services, particularly for newcomers and marginalized populations (20–22).

Participants shared that even when multiple resources, support, and services are available, there is often a general lack of public awareness about these resources, affecting not only newcomers who face language and cultural barriers but also all potential users of healthcare systems and healthcare providers. In these cases, the role of navigators, whose primary aim is to identify and coordinate resources, care, and treatment and to ease the client's journey through the healthcare system, becomes invaluable. This emphasizes the importance of navigation support, particularly for individuals facing new or complex health issues and those in socially disadvantaged groups (3, 15–23).

A key finding is that, regardless of their specific role, navigators serve as crucial links between their clients and the healthcare system, helping to make the healthcare journey less daunting and promoting positive, more inclusive health-seeking behaviors. We confirmed that the scope of the navigator role is broad and multifaceted, varying based on the specific conditions being addressed and the type of navigation services offered (4, 19, 22–25). Informal navigators may assist with tasks such as accompanying individuals to doctors' appointments or providing interpretation and translation services, while professional navigators may offer specialized support for specific conditions, such as pediatric care coordination, cancer care, dementia, or diabetes. This diversity in navigator roles highlights their contribution to overcoming the barriers and complexities of the healthcare system (4, 22, 24, 25).

Many of the participants in the Navigator group shared that their motivation to become navigators stemmed from an intrinsic passion for helping others, especially those who have become discouraged with the complexities of the healthcare system. In such instances, the relatively informal yet respectful relationship between the navigator and client—built on mutual trust, empathy, and cultural sensitivity—enables the navigator to act as an advocate, educator, mediator, supporter, and even a friend, helping clients to “connect the dots” and access the care they need. The findings of this study align with a study by Phillips et al. (26) that explored navigators’ reflections on the navigator-patient relationship, describing the navigator role as providing motivational support throughout the patient's clinical care (26–28).

Navigators play an important role in addressing additional issues of racism and discrimination within the healthcare system by reducing barriers that prevent access to quality healthcare among immigrant, marginalized, and low-income populations (24). One participant shared an experience of misdiagnosis in an urban Alberta hospital that she felt was the result of discrimination based on her being a member of a visible minority group, highlighting systemic issues that resulted in a stroke in her case. The same participant shared numerous instances of being denied the chance to accompany her mother as an interpreter when the used phone language line was unsuccessful due to cultural misunderstanding or missed situational details. These situations underscore how healthcare navigators can help avoid such problems, especially for socially disadvantaged groups.

The navigators highlighted the importance of incorporating formal interpretation and translation training into the navigator role, which would greatly improve the healthcare experience for clients who require these services. Language and communication barriers, as highlighted in one participant's story, pose substantial challenges to healthcare delivery, especially among newcomers in Alberta. Existing language tools, such as language lines, often fall short in addressing these barriers, as they may not account for the sociocultural complexities of clients, including dialects, hierarchical structures, and gender dynamics.

An important finding was the personal connection between the navigators and their clients, noting that this relationship can significantly enhance the client's healthcare experience. The participants in the Navigated group also highlighted the value of sharing one or more similar characteristics with their navigators, whether it was language, culture, or a similar health condition. They confirmed that when trying to understand and access healthcare services and resources after a diagnosis, it was important to them that their navigator had a strong knowledge of the healthcare system, experience related to their health condition, and familiarity with the pathways available for treatment. Even with minimal interventions, the majority of the participants in the Navigated group expressed satisfaction with the services they received from their navigator, noting that the latter’s services were instrumental in improving their overall health outcomes, including mental health.

This study highlights the vital role healthcare navigation has in bridging the gaps between patients and community members and the complexities of healthcare systems, and further emphasizes how shared culture, language, and lived experience can significantly enhance the quality of navigation services, facilitating the provision of effective person- and family-centered care. The scope of navigation services in Alberta is constantly expanding, with more specialized roles emerging. This study also reemphasizes the need for ongoing development and enhancement of navigation services and navigator training in Alberta. Evidence from our literature review (available elsewhere) identified key reviews describing several programs tailored to the development of navigation services for specific health conditions (3, 16–19, 21).

## Strengths and limitations

A strength of this study is the strong connections our team members had with their respective communities, which proved instrumental in achieving more inclusive and representative participant recruitment. This was especially important when we faced hurdles such as difficulties recruiting through healthcare and community organizations and a setback caused by social media spammers that led to the closure of our Qualtrics survey. The deep-rooted community ties of our group members allowed us to navigate these obstacles and successfully achieve our recruitment goals. Furthermore, the linguistic and cultural diversity within our team also significantly enhanced the data collection and analysis process. Team members fluent in Punjabi provided interpretation during one focus group, enabling seamless communication and interaction with the participants. A comprehensive translation of the focus group transcript allowed us to capture emotional nuances and details of the participants’ stories, resulting in richer and more accurate data for our analysis.

The majority of the participants were from Calgary, with some from Edmonton. This study had limited representation from rural Alberta, with only one participant in the Navigated group and three in the Navigator group from that region. The member-checking focus groups (REFLECT) validated the findings, adding to the robust study design (12).

This study encountered several limitations that should be considered when interpreting the results. A significant limitation was the tight timeline to complete participant recruitment at the COLLECT stage (1.5 months). Some organizations required lengthy administrative procedures to obtain permission to recruit participants, which limited the number of participants available to recruit. Organizational policies surrounding confidentiality and privacy were also major obstacles to recruiting participants in the Navigator group. This study was limited by the overrepresentation of female participants, individuals aged 31–50 years, and those residing in urban settings. Women may be more engaged both as users and providers of navigation services due to their greater involvement in healthcare and caregiving, while recruitment pathways may have disproportionately reached this group. Similarly, adults in midlife may be more visible in navigation roles, whereas the perspectives of younger and older populations remain underrepresented. Finally, the predominance of urban participants limits insights into the distinct navigation challenges faced by rural and remote populations. Together, these factors constrain the generalizability of findings and highlight the need for further research with more diverse participant groups.

## Conclusion

The study highlights the need for ongoing efforts to formalize and expand healthcare navigation services in Alberta with a focus on personalizing care to support vulnerable groups, such as those requiring cancer care, disability care, and mental health services. This can best be achieved through the integration of patient engagement into learning health systems. Learning health systems should provide a culturally sensitive, person-centered healthcare navigation experience, broaden the scope and availability of navigation services across various health conditions, and increase public awareness through targeted strategies and outreach. Continuous evaluation of these programs, using a learning health system approach, is vital to adapt to evolving healthcare needs and ensure effective patient-centered service delivery. The findings of this study may be considered relevant to a broader audience in Canada and globally, as they address current issues, such as gaps and best practices in navigating complex healthcare systems, and provide an exploration of navigation services from the perspective of immigrants, which is a particularly relevant topic given ongoing global migration (29, 30). This study provides a framework for larger, long-term research aiming for a more comprehensive scan of navigation programs in Alberta, including rural parts of the province, that could lead to the creation of a Directory of Healthcare Navigation Programs available to Albertans. Further research, including broader geographical representation of Albertans with diverse lived experiences, will be beneficial to inform the ongoing research on healthcare navigation delivery and training.

## Data Availability

Due to privacy and confidentiality considerations, we are unable to share the raw data. Nevertheless, we are committed to transparency and will consider sharing anonymized data upon reasonable request and in accordance with data sharing policies.
