# Supplementary material for: From experience to a learning health system: peer-to-peer perspectives and implications for healthcare navigation in Alberta, Canada
Source: Front Health Serv. 2025 Oct 17;5:1642188. doi: 10.3389/frhs.2025.1642188 (PMC12575376; doi:10.3389/frhs.2025.1642188)
Supplement: Supplementary file 1 [file Table1.docx]

Understanding healthcare navigation in Alberta: What is important to navigators and to people who have been navigated in the Alberta healthcare system?

**COLLECT Focus Group Guide**

**(Navigated)**

| **Time (will vary)** | **Activity** |
| --- | --- |
| 10:00-10:10 | Welcome, Introductions, Administrative  -Thank people for coming  - Land Acknowledgement  - Establish some ground rules for your conversation (including consent to record the meeting)  - Team introduction (ask everyone to introduce themselves and explain the roles they will be assuming for the session)  -Ask patient participants to introduce themselves and answer an ice breaker question in one sentence “What is your favourite dessert?”  -Ask participants for their consent to record today's meeting and remind them that they can withdraw ant time during our meeting |
| 10:10-10:15 | Purpose of this focus group:  -Share their perspective and experiences on health navigation by answering our questions |
| 10:15-10:20 | Summarize how we developed research question:  - Give a brief research summary  -Results from SET group meeting |
| 10:20-11:40 | Focus Group Question Guide  - How did you come to know about patient navigation services? How was your experience accessing the services?​  (Was that easy/difficult?)  - What are your experiences being navigated through the healthcare system in Alberta?​  (booking specialists appointments, tests, going for appointments etc.)  - How important is it for you to get this help to navigate through Alberta healthcare system?​  (getting to appointments, booking tests, specialist’s appointments, language support etc.)  - What were your challenges and advantages in using patient navigation services?  - What is important to you in your ideal navigators?​  (age, gender, shared lived experience, cultural background, language, experience and understanding of health care systems, etc.)  - What language was used to communicate with your Navigator? ​  (Was it your own language? Did you use an interpreter in the navigation process?)​  - Which aspects or services of patient navigation are most helpful to you?  (medical information, care connection with healthcare providers, emotional support, other)  - Is there anything that could have improved your navigation experience? |
| 11:40-11:50 | Summarize the information collected |
| 11:50-12:00 | Wrap up and discuss the engagement   - - - Tell us what you discovered today through this group conversation?     - What will you take away from this session? |
