# Supplementary material for: From experience to a learning health system: peer-to-peer perspectives and implications for healthcare navigation in Alberta, Canada
Source: Front Health Serv. 2025 Oct 17;5:1642188. doi: 10.3389/frhs.2025.1642188 (PMC12575376; doi:10.3389/frhs.2025.1642188)
Supplement: Supplementary file 2 [file Table2.docx]

Understanding healthcare navigation in Alberta: What is important to navigators and to people who have been navigated in the Alberta healthcare system?

**COLLECT Focus Group Guide**

**(Navigators)**

| **Time (will vary)** | **Activity** |
| --- | --- |
| 10:00 – 10:10 | Welcome, Introductions, Administrative  - Welcome participants and thank them for their participation.  - Land acknowledgement.  - Consent to participate  - Establish some ground rules for the meeting including (emphasizing a mutually respectful space without any bias and reminding participants to only share information they are comfortable with sharing).  - Introductions – a) First the Navigators research team will introduce themselves and their roles, then answer the ice breaker question “what are your plans for the summer break this year?” b) Then the participants will introduce themselves, then answer the ice breaker question “what are your plans for the summer break this year?”  - Obtain consent for recording meeting, and remind participants that they can withdraw consent at any time |
| 10:10 – 10:15 | Purpose of this focus group:  - The purpose of this focus group is for you to share your experiences and perspectives as a health navigator who has provided navigation services for clients/people in the Alberta Health care system. We have a couple of questions prepared, and we hope to get your answers and insights on these questions. |
| 10:15 – 10:20 | Summarize how you developed research question and PaCER process:  - Give a brief background on the importance and rationale for our research: limited amount of adequate literature on health navigation in Alberta.  - Explain the PaCER process, and how we reviewed previous pacer projects, research articles, and available grey literature on health navigation.  - We arrived at a draft research question, which was finally restructured to the current research question based on the feedback we received during our SET discussion meeting.  - State the research question.  - Explain that their input is valuable and will greatly contribute to our research. |
| 10:20 – 10:40 | Focus Group Question Guide:  1.What motivated you to become a health navigator?  2.Can you describe your role as a health navigator in Alberta?  - Probes – what specific populations do you serve, how are you described? Are you paid?  3.What resources or supports have you found most useful in your role as a health navigator?  - Probes – type of training, support for compassion fatigue, mental health, etc.  4.What are some Challenges have faced in your role as a health navigator?  - Probes - Communication with navigated, experience collaborating with other health care professionals or organizations while navigating patients through the health care system?  5.What are some positive experiences you have encountered in your role as a health navigator?  6.What shared characteristics such as (language, culture, faith, lived experience with health conditions, gender, and age) are important for success in health navigation?  - Probes – How do these characteristics impact your role as a health navigator?  7.What improvements or changes do you believe are needed to enhance the effectiveness of health navigation services in Alberta?  - Probes – Which areas or specific populations do you believe needs more navigation services? |
| 10:40 – 10:50 | Summarize the information collected:  - Summarise key points discussed during the focus group. |
| 10.50 - 12.00 | Wrap up and discuss the engagement  - Tell us what you discovered today through this group conversation?  - What will you take away from this session?  - Do you have additional thoughts or suggestion for us?  - Share that after the data has been analyzed and themed, the participants will be invited to the REFLECT focus group.  - Thank Participants for their time and input.  - Share the demographic survey.  - Provide information on how participants can contact us for further feedback and follow-up. |
