# Supplementary material for: From experience to a learning health system: peer-to-peer perspectives and implications for healthcare navigation in Alberta, Canada
Source: Front Health Serv. 2025 Oct 17;5:1642188. doi: 10.3389/frhs.2025.1642188 (PMC12575376; doi:10.3389/frhs.2025.1642188)
Supplement: Supplementary file 3 [file Datasheet1.pdf]

Has someone ever helped you navigate your way through the Alberta healthcare system?

Are you 18 years or older?
Are you living in Alberta?

We are Patient and Community Engagement Research (PaCER) student researchers looking to understand what is important to people who have been navigated through the Alberta healthcare system.

What will you do?

Share your thoughts and experiences in

- **One online focus groups** of 2 hours
  - **Tuesday, May 14**, 2024. 10:00am to 12:00pm
  - **Wednesday, May 22**, 2024. 6:00pm to 8:00pm
  - **Saturday, May 25**, 2024. 5:00pm to 7:00pm

Or

- **One individual interview** of 1 hour **(online or in-person)**

Interviews may be available also in the following languages: Arabic, Azerbaijani, Dari, Hindi, Mandarin, Pashto, Punjabi, Russian, Spanish, and Urdu.

To participate go to
https://survey.ucalgary.ca/jfe/form/SV_0Dmrma6qHfUaG6W_

If you have any question please email
fakhriyya.aghabayli@ucalgary.ca

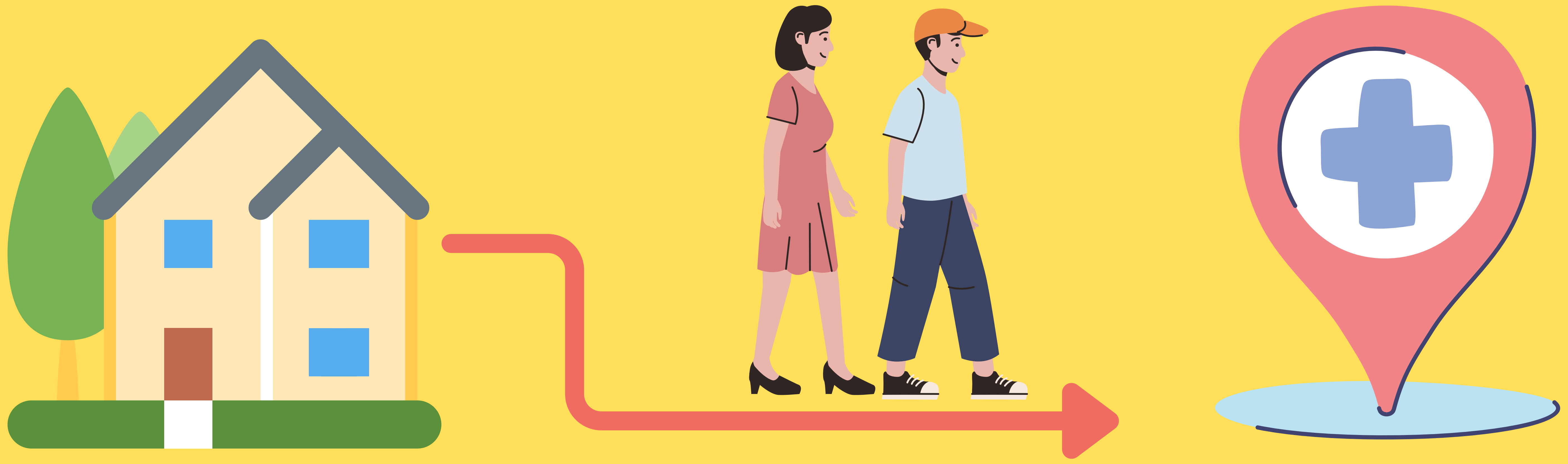

UNIVERSITY OF CALGARY
Patient and Community Engagement Research

AbSPORU
Alberta SPOR SUPPORT Unit

This study has been approved by the University of Calgary Conjoint Health Research Ethics Board **(REB24-0389)**.
