# Supplementary material for: From experience to a learning health system: peer-to-peer perspectives and implications for healthcare navigation in Alberta, Canada
Source: Front Health Serv. 2025 Oct 17;5:1642188. doi: 10.3389/frhs.2025.1642188 (PMC12575376; doi:10.3389/frhs.2025.1642188)
Supplement: Supplementary file 4 [file Datasheet2.pdf]

Do you have an experience helping people to navigate the Alberta healthcare system?

Are you

- **18+ years**
- **living in Alberta?**

Join us for a Zoom focus group on:

Friday, May 17, 2024 at 5-7 pm; MT

or

Saturday, May 25, 2024 at 3-5 pm; MT

or

Wednesday, May 29, 2024 at 6-8 pm MT

If so, we want to hear from you!

Share your experiences & thoughts in an online or in-person

- **focus group (≈2 hrs)**

or

- **an individual interview (≈1hr)**

We are:

Patient and Community Engagement Research (PaCER) Student Researchers looking to...

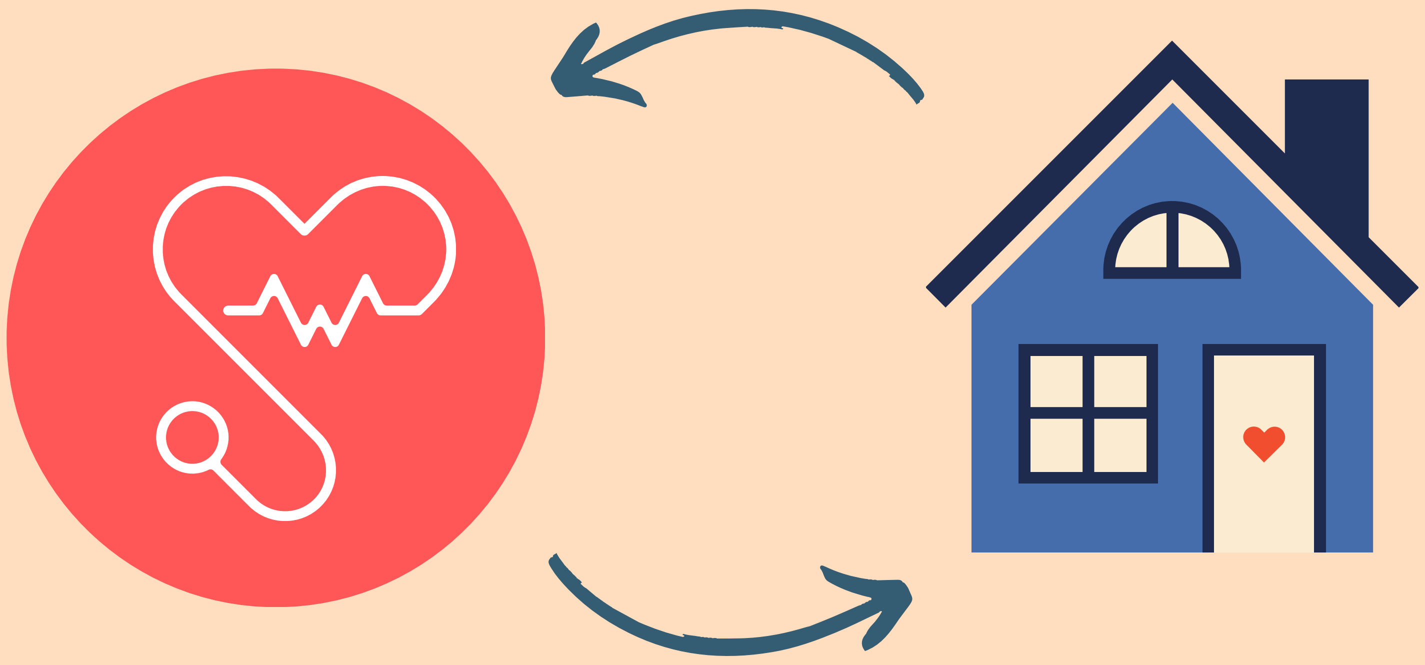

"...Understand the experiences and perspectives of health navigators and those who have been navigated in the Alberta healthcare system"

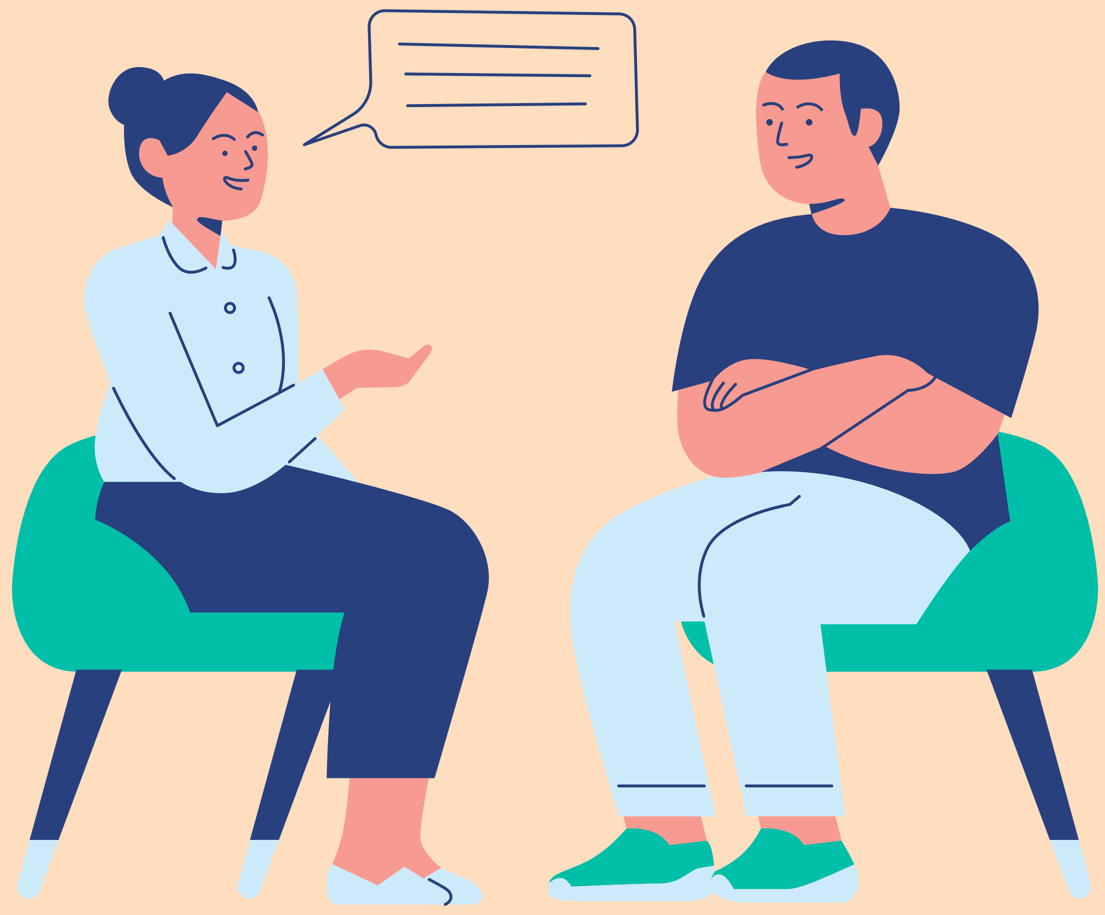

Please use the link here or the QR code given below to sign up

or reach out to our team for more information: huda.khan@ucalgary.ca

AbSPORU
Alberta SPOR SUPPORT Unit

UNIVERSITY OF CALGARY
Patient and Community Engagement Research

This study has been approved by the University of Calgary Conjoint Health Research Ethics Board- REB24-0389
